# Crossing the Gap: Older Adults Do Not Create Less Challenging Stepping Stone Configurations Than Young Adults

**DOI:** 10.3389/fpsyg.2020.01657

**Published:** 2020-07-10

**Authors:** Amy M. Jeschke, Annemieke M. M. de Lange, Rob Withagen, Simone R. Caljouw

**Affiliations:** Department of Human Movement Sciences, University of Groningen, University Medical Center Groningen, Groningen, Netherlands

**Keywords:** active aging, affordances, gap crossing, built environment, multi-target stepping, physical activity designs, stepping stone configurations

## Abstract

**Background:**

Despite the known health benefits of physical activity, the number of older adults exercising regularly remains low in many countries. There is a demand for public open space interventions that can safely train balance, muscular strength, and cardiovascular fitness. In this participatory design study, older adults and young adults were to create their own stepping stones configurations. We provided them with six stepping stones, and examined the gap widths that each group of participants created and how they used the configurations.

**Results:**

The created absolute gap distances by the older adults were smaller than those of the younger adults. Yet, the amount of challenge (in terms of the created gap widths relative to a person’s estimated stepping capability) did not significantly differ between the young and older adults. Furthermore, both groups created non-standardized stepping stone configurations in which the number of different gap widths did not significantly differ between the young and older adults. Interestingly, while using their personalized design, older adults made significantly more gap crossings than younger adults over a given timespan. This finding tentatively suggests that personalized design invites physical activity in older adults.

**Conclusion:**

The present study demonstrated that older adults are not more conservative in designing their own stepping stone configuration than young adults. Especially in light of the public health concern to increase physical activity in seniors, this is a promising outcome. However, field tests are needed to establish whether the older adults’ stepping stones designs also invite physical activity in their daily environment.

## Introduction

More people than ever are living long lives (World Health Organization [WHO], 2018). When we look at older age groups, movements become slower, muscle strength decreases, eyesight reduces, and there is an increased risk of falls (Osoba et al., 2019). To stay healthy and to maintain the ability to perform everyday activities independently, older adults are recommended to be physically active (World Health Organization [WHO], 2011). Despite the known health benefits of physical activity (World Health Organization [WHO], 2011), the number of (older) adults exercising regularly remains low in many countries (Hallal et al., 2012; World Health Organization [WHO], 2014). This means that increasing physical activity in seniors is a public health concern.

Over the last decades outdoor exercise areas have been frequently implemented to safely train balance, muscular strength, and cardiovascular fitness (Arena et al., 2017; Kershaw et al., 2017). Yet, there is a lack of empirical research with respect to both the usefulness and the appropriateness of the equipment that is used in these areas (Chow, 2013; Chow et al., 2017; Kershaw et al., 2017; Chow and Wu, 2019). To our knowledge, only one recent study provided empirical data regarding the use of such equipment (Chow and Wu, 2019). It was found that older adults used the equipment, but only for very short amounts of time. Moreover, the equipment was frequently used in a way that was not intended by the manufacturers. In fact, a panel of experts indicated that the observed alternative behaviors on this equipment were often not safe (Chow and Wu, 2019). Hence, there seems to be a need for better-fit designs to make outdoor equipment more inviting and better useable for older persons. The present study aims to contribute to this.

Ever since its introduction, the concept of affordances has been used to both understand the environment we act in Heft (1988), Beek and de Wit (1993), Withagen et al. (2012), Withagen and Caljouw (2016) and to build it Rietveld (2016). The concept of affordances was introduced by Gibson to refer to the action possibilities in an environment of an agent (Gibson, 1979). For young adults, a puddle affords jumping over and an aperture between sliding doors affords squeezing through (Davids et al., 2016). Crucially, affordances exist by virtue of the relationship between the properties of the environment and the action capabilities of the actor (Gibson, 1979). For instance, whether a gap is crossable for a senior (and how challenging it is for her) depends on the gap width relative to the senior’s maximum stepping distance. It is therefore essential to design environments for physical activity that offer the right affordances, so that they are accessible, inviting, functional, and useable, also for seniors with a contracting range of action possibilities (Withagen et al., 2012; Davids et al., 2016).

In keeping with the participatory design techniques (Francis, 1988), Jongeneel et al. (2015) recently conducted a study on the affordances of stepping stone configurations. In their study children were to create their own playing area by placing six jumping stones. As can be expected from an affordance perspective, it was found that the created gap widths matched the children’s action capabilities. Results also revealed that most children created messy structures with a variety of gap distances (Jongeneel et al., 2015). These non-standardized stepping stones configurations of the children were a far cry from the symmetric figure eight configurations that can be found in the celebrated playgrounds of Aldo van Eyck (Jongeneel et al., 2015; Withagen and Caljouw, 2017). The study of Sporrel et al. (2017a) further investigated the preferences and play behavior of children by presenting them with a non-standardized and a standardized jumping stones configuration. It was found that children liked the non-standardized playground better and spent more time playing on it compared to the standardized playground. This indicates that children indeed created the design they like best.

Stepping stones playgrounds are arguably also relevant exercise areas for older adults as multi-target stepping is recommended for balance training in older adults (Sherrington et al., 2008; Okubo et al., 2017). Meta-analyses indicated that an effective fall prevention training must provide a challenge to balance (Sherrington et al., 2008; Okubo et al., 2017). Recommended activities are, for example, moving the center of gravity reducing the base of support, and decreasing the use of arm support (Sherrington et al., 2008). Moving from one stone to another requires adequate weight-shifting and foot placement, both important capacities to regain balance after disequilibrium. Hence, there are promising indications that stepping stones configurations can be used as a work-out area for older adults to train balance and foot-placement in order to prevent falling.

The aim of the current study was to examine what kind of stepping stones configurations seniors create for themselves and how they use it. Functional declines in older adults may lead to fear of falling and appropriate increased caution to prevent falls (Todd and Skelton, 2004; Scheffer et al., 2008; Osoba et al., 2019). Therefore, it is expected that older adults will create smaller gap widths, reflecting the age-related change in action-capabilities, and also less challenging gap widths relative to their action capabilities compared to younger adults. To evaluate the challenge of the created gap distances, we scaled the created distances to the participant’s (estimated) maximum stepping distance. We also examined challenge by exploring the amount of variation of the created gap widths. Arguably, a design with a great variety of gap widths is more challenging than a more predictable standardized design with equal distances, because one needs to adjust every next step when stepping from stone to stone (Sporrel et al., 2017b). We hypothesized that older adults would opt for a more standardized design than young adults, taking into account the common fear of falling and age-related declines in perception and action (Todd and Skelton, 2004; Scheffer et al., 2008; Osoba et al., 2019). After designing their own configuration for training multi-target stepping, participants were asked to actually play on their own design for 2 min. We evaluated the number of gaps and the distance crossed to explore how the young and older adults used their own design.

## Materials and Methods

### Participants

Twenty-five young adults ranged from 19 to 30 years old (*M* = 23.28, *SD* = 3.05) and 24 older adults ranged from 61 to 78 years old (*M* = 96.79, *SD* = 4.71) participated in this study. Within the young and older groups of adults, participants were, respectively, 40% and 50% female. All participants were community dwelling and reported to be healthy and able to walk outdoors without walking aids. The study was approved by the Institutional Ethics Committee (ECB/2016.02.07_1) and conducted in accordance with the Declaration of Helsinki. Informed consent was obtained from all individual participants included in the study.

### Design and Procedure

The study was divided into three parts. In the first part, anthropometrics and the estimated and actual action capabilities of the participants were measured. In the second part, the participants were asked to design their own stepping stones configuration, and in the third part the participants were asked to use the configuration. The experiment took place indoors in an empty classroom of the University of Groningen. Participants were asked beforehand to wear comfortable flat-soled shoes.

#### Anthropometrics and the Estimated and Actual Action Capabilities

We first measured body height while the participant was standing straight against the wall. To determine the participant’s leg length, we then measured the height of the participant while she was sitting straight on a chair. Leg length was computed by subtracting the difference between sitting height and chair height from the standing height.

Second, we measured the participants’ estimated and actual maximum stepping distance. Two circular carpet tiles were used that were identical to the stepping stones used for designing the configurations. One stepping stone was placed at a fixed location, the other stepping stone could be placed at different distances. The experimenter demonstrated what was meant by stepping. To that end, the experimenter started with both feet next to each other at the edge of the first stepping stone. She made a step and after landing the leading foot on the second stone, she removed the trailing foot from the first stone and placed it next to the leading foot on the stepping stone. Both feet were placed entirely on the next stepping stone. After the demonstration the participant was instructed to stand on the fixed stepping stone with both feet at the edge, and to stay in that position during the estimation trials – obviously stepping was not allowed. To assess the participants’ estimation of the maximum stepping distance we used a modified staircase method. To prevent the error of anticipation (Woodworth and Schlossberg, 1954), which is considered a limitation of this method, we performed an ascending and a descending staircase trial. In the ascending condition the movable stepping stone was initially placed at a distance of 50 cm from the fixed stepping stone for the young adults. For the older adults the initial gap distance was set at 25 cm, as they generally have a smaller step capacity than young adults (Osoba et al., 2019). The stepping stone was moved away from the participant in steps of 5 cm. Each time the stone was moved to a new position the participant was asked to estimate (answering “yes” or “no”) whether she could cross the gap by stepping. The experimenter increased the gap distance in steps of 5 cm until the participant judged the gap to be too wide. After a negative response the gap was decreased with one step of 10 cm (or more steps if, again, perceived not crossable). This procedure was repeated, i.e., the distance was increased with 1-step after a positive response and decreased with 2-steps after a negative response and increased again until the maximum perceived distance was reached for the third time and written down. In the descending condition, the moveable stone was initially placed at a distance of 150 cm for both the young and older adults. The experimenter decreased the gap with 5 cm per time until the participant estimated the gap small enough to cross with a step. At that point, the gap distance was increased with 10 cm (or more steps if perceived crossable). As in the ascending condition this process was repeated twice to reliably determine the estimated maximum stepping distance. So, we used a 1-up 2-down adaptive staircase procedure for the ascending trial and a 1-down, 2-up adaptive staircase procedure for the descending trial. The average of both estimations was taken as the maximum perceived crossing distance^1^.

Lastly, the actual maximum stepping distance was measured by asking the participants to make an overground step as large as possible. The experimenter first demonstrated how to execute the maximum step. She was standing with both feet next to each other behind a line, made a large step forward, and, after the double support phase, placed the trailing foot next to the leading foot. The participants were given three attempts; the largest maximum stepping distance reached in three attempts was notated as the maximum stepping distance. Maximum stepping distance was measured from the starting line to the heels of the feet after stepping.

#### Designing the Training Configurations

Participants were instructed to create their own stepping stones configuration to practice stepping. They were asked to create their configuration with six stepping stones and subsequently use it for a set amount of time. The stepping stones were circular and had a diameter of 50 cm. To reduce the fall risk, the stones had a negligible height. They were made from carpet with a rubber anti-slip mat fixed to the bottom to prevent slipping. In the classroom, an area (5 m × 7 m) was marked with tape, and in the middle of this area one stepping stone was fixed. The participants had to create their configuration within the area. The participants were free to place the remaining five stepping stones around the fixed stepping stone, as long as they could step from one stone to the other without touching the ground. To experience whether the constructed configuration was in line with her desires, the participant was allowed to step from one stone to the other and to walk freely over the ground during this phase. There was no time limit given for constructing the design. After the participants were finished, the distances between the stepping stones were measured with a ruler.

#### Stepping Behavior

Participants were asked to step from one stone to another for a fixed time period of 2 min in the configuration they had created. Stepping behavior was video recorded using a digital camcorder (GoPro Hero4 Silver). The starting point for each participant was the stepping stone that was fixed in the middle of the room. The participants were instructed to step from one stone to the other without touching the ground, in any direction and speed desired. The start signal was given to the participant after a countdown from three, after which video recording was started. Based on the video recordings, the crossed gaps and the number of times these gaps were crossed were determined.

### Data Analysis

By placing six stones, one necessarily creates 15 gaps. However, not all of the gaps are functional gaps to the participant as they cannot always be crossed. We used the participant’s stepping behavior to determine which gaps were crossed, and only these gaps were included in the analyses (see also Jongeneel et al., 2015). For each design, we determined the minimum, maximum and mean width of the functional gaps created. To evaluate the challenge in the design, we followed Jongeneel et al. (2015) and divided the gap width measures of each configuration by the individual’s estimated maximum stepping distance. The rationale for using the estimated stepping distance rather than the actual distance in calculating the challenge ratios was that the former is arguably leading in determining whether a participant decides to cross a gap. Hence, when a participant crosses gap widths that are close to her estimated maximum stepping distance it is, in our view, fair to conclude that this participant opts for challenging gaps. To determine the variation in the created gaps we calculated the number of different gap distances created by means of a hierarchical cluster analyses using the furthest neighbor method, setting the cut-off point at 10% of the individual’s mean gap width (Jongeneel et al., 2015). This means that within each of the created clusters, the differences between the gap widths did not exceed 10% of the average gap width of that person.

Statistical analyses were performed using the software IBM SPSS Statistics software (Version 24.0 for Windows, SPSS Inc., NY, United States). Normality checks showed that most data were normally distributed, except for data regarding the numbers of gaps crossed and the numbers of functional gaps created. A Two-Way Mixed ANOVA was used to compare the estimated and actual stepping distance between young and older adults. Furthermore, paired sample *t*-tests were used to test for differences between the age groups in the created mean, minimum and maximum gap widths and the ratios of these widths with the participant’s estimated stepping distance. Wilcoxon rank-sum tests were used to compare the number of gaps crossed and the number of different gap widths between the young and older adults. The level of significance was set at 0.05.

## Results

**FIGURE 1:**
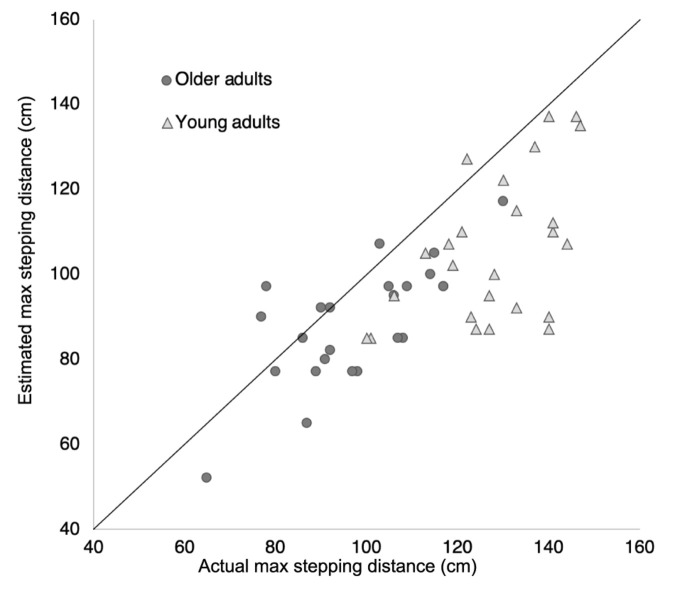
The estimated and actual maximum stepping distance displayed for the young (triangles) and older (circles) participants. Data points above the diagonal indicate overestimations.

Table 1 and Figure 1 present the estimated and actual maximum stepping distance of the young and older participants. The Age (young vs. older adults) × Condition (estimated vs. actual capability) ANOVA revealed significant main effects of Age [*F*(1, 47) = 37.60, *p* < 0.001, η*_*p*_^2^* = 0.44], indicating that older adults had a smaller estimated and actual stepping distance than the young adults. Most data points in Figure 1 are below the diagonal, indicating that most participants underestimated their maximum stepping distance. Furthermore, the ANOVA revealed a significant main effect of Condition [*F*(1, 47) =33.44, *p* < 0.001, η*_*p*_*^2^ = 0.42] and a significant Age x Condition interaction effect [*F*(1, 47) = 10.45, *p* < 0.002, η*_*p*_*^2^ = 0.18] indicating that the young adults underestimated their maximum stepping distance to a larger extent than older adults.

**TABLE 1:** Means (Standard Deviations) of leg length, the actual and the estimated maximum stepping distance for the young and older adults in cm.

	Young adults	Older adults
Leg length	87.1 (7.1)	87.9 (5.5)
Actual max step distance	128.0 (13.5)	98.0 (15.5)
Estimated max step distance	111.4 (16.9)	93.3 (14.5)

### Design Characteristics

**FIGURE 2:**
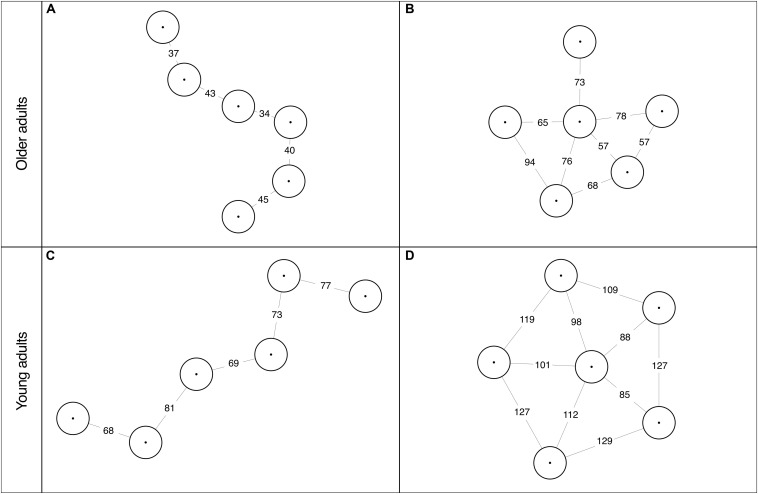
Four examples of the created stepping stone configurations of two older adults **(A,B)** and two young adults **(C,D)**. Gap widths are presented in cm for the gaps that were crossed by the participant.

Subsequently, we examined the characteristics of the stepping stones configuration created by the young and older adults. Characteristics of the created stepping stones configurations for each participant can be found in Table A1. Figure 2 shows examples of the configurations created by two older adults (upper row) and two young adults (bottom row). A Wilcoxon rank-sum test revealed that the number of functional gaps created in the configurations did not differ significantly (*Ws* = 582, *Z* = −0.88, *p* = 0.38, *r* = −0.13) between the young (*Mdn* = 6, range 5–14) and older adults (*Mdn* = 6.5, range 5–10). As expected, older adults created configurations with smaller gap widths than the young adults (see Table 2). Independent *t*-tests confirmed that the two groups differed significantly from each other in the created minimum [*t*(47) = 3.39, *p* < 0.01], mean [*t*(47) =3.93, *p* < 0.001] and maximum [*t*(47) =7.24, *p* < 0.001] gap widths. Interestingly, we found that the challenge ratios for the maximum gap width [*t*(47) = 1.13, *p* = 0.26], the mean gap width [*t*(47) = 1.80, *p* = 0.08] and the minimum gap width [*t*(47) = 1.82, *p* = 0.076] were not significantly different between the age groups (see Table 2)^2^. As mentioned in the introduction, challenge is also realized by creating a variety of gap widths, as it requires the participant to adjust its movements to the different gap widths in the environment. A Wilcoxon rank-sum test revealed that the number of different gap widths created, determined by the hierarchical cluster analyses, did not differ significantly (*Ws* = 561.5, *Z* = −1.34, *p* = 0.18, *r* = −0.19) between the young (*Mdn* = 3, range 2–5) and older adults (*Mdn* = 3, range 1–7). Only one out of 49 participants created a configuration in which the crossed gaps had the same widths. Most older adults (20 out of 24) created a design with at least three different gap widths. The training area of one older adult consisted of even 7 different gap widths that were crossed, ranging from a small gap distance of 43 cm to gap distances that were larger than his initially estimated maximum distance to step. Hence, if older adults are the architect of their own configuration, the vast majority of them create a challenging design with a variety of gap widths.

**TABLE 2:** Means (Standard Deviations) of the minimum, mean, and maximum created (and crossed) gap widths and the corresponding ratios between these gap widths and the participant’s estimated maximum stepping distance.

	Young adults	Older adults	*p*-value
Min gap width (cm)	70.60 (23.23)	50.71 (17.33)	*p* = 0.001*
Ratio min gap width	0.63 (0.20)	0.54 (0.15)	*p* = 0.076
Mean gap width (cm)	84.45 (20.29)	63.02 (17.72)	*p* < 0.001*
Ratio mean gap width	0.76 (0.17)	0.68 (0.15)	*p* = 0.078
Max gap width (cm)	98.44 (18.88)	77.50 (21.54)	*p* = 0.001*
Ratio max gap width	0.89 (0.17)	0.83 (0.20)	*p* = 0.264

*Significance p = 0.001.

### Stepping Behavior

Based on the video-recordings of the stepping behavior during the play phase we determined the total number of gaps crossed and the total gap distance covered (see Table 3). The Wilcoxon rank-sum test revealed that the older adults made significantly more gap crossings than the young adults (*Ws* = 480, *Z* = −2.90, *p* = 0.004, *r* = −0.41). The total gap distance covered was not significantly different between the two age groups [*t*(47) = 0.52, *p* = 0.61]. This indicates that older adults, with their reduced stepping capacities, were at least as active as young adults when crossing gaps in their own configuration.

**TABLE 3:** Median (Interquartile Range) of the number of executed steps and mean (Standard Deviations) of the distance covered by participants during the 2 min step time.

	Young adults	Older adults
Number of gaps crossed	51 (41–59)	66 (54–75)
Total gap distance covered (m)	40.77 (9.14)	39.24 (11.59)

## Discussion

The present study examined whether older adults and young adults differ in the stepping stones configurations they create for themselves, focusing on both the distances between the stones and the variety of gap widths. In addition, we explored how the participants used their own configurations. To that end, we determined the maximum, mean and minimum gap widths in the participant’s stones configuration. To examine whether these gap widths were challenging for the participant we computed ratios – we divided these widths by the estimated maximum stepping distance of the participant. In addition, we checked whether the stepping stone configuration was standardized (equal distances between the stones) or whether a variety of gaps was created.

Contrary to our hypothesis, we found that older adults did not create less challenging stepping stone configurations than the young adults. Although we found, as expected (Osoba et al., 2019), that older adults created smaller gap widths than young adults, the ratios of the maximum, mean, and minimum created gap width with the participant’s estimated stepping distance were not significantly different between the age groups. In other words, the amount of challenge did not significantly differ between the young and older adults. Furthermore, when evaluating the variety of gap widths, both groups created an non-standardized stepping stone configuration in which the number of different gap widths did not significantly differ between the young and older adults. Such non-standardized configurations also underpin the choice for challenge, as they demand more balance control and action preparation than standardized configurations (Sporrel et al., 2017b).

However, the fact that the challenge ratios were close to 1 for the maximum gap widths might also have to do with our method to determine the estimated and actual maximum stepping distance. In measuring these distances, the participant was to step from a standing position and was only allowed to lift the trailing foot after the leading foot was placed (i.e., after double support). During the play phase, on the other hand, participants could cross gaps while moving. This allowed them to use some momentum, implying they could cross wider gaps. Moreover, testing the participant’s estimated and actual maximum stepping distance at the start of the experiment might have primed our participants to create a challenging design. After all, they were aware of their capacities before they designed their configurations. However, our most important finding relates to the observation that we did not find significant differences in the challenge ratios between the younger and older adults. That is, we found *no* indication for the proposition that older adults are more conservative in designing their stepping stone configurations. In subsequent research it would be interesting to examine whether we can replicate this finding when real stones rather than flat carpet tiles are used. It might be that introducing height may lead to a more cautious design by the older adults, because of the larger risk of falling.

When evaluating stepping behavior, the total gap distance covered was not significantly different between the two age groups – the older adults made smaller, but significantly more gap crossings than the young adults. This tentatively suggests that personalized design of the environment could perhaps be a method to counteract the earlier reported physical inactivity in (older) adults (Hallal et al., 2012; World Health Organization [WHO], 2014). Indeed, to invite physical activity, the affordances of an installation should be in place for the intended users (Withagen et al., 2012). As mentioned at the outset of the paper, affordances exist by virtue of the physical dimensions of the environment relative to the action capabilities of the agent (Gibson, 1979). That is, the body should be taken into account when designing the environment, and arguably the intended user is best equipped in doing so. Indeed, in keeping with the study on how children design their stepping stones configuration (Jongeneel et al., 2015), we found that also older adults are capable of designing a configuration that matches their abilities and which gives rise to physical activity.

Granted, further studies are needed to test whether the design of the older adults indeed invites physical activity when they are installed in their daily environment. After all, the present study cannot rule out that older adults might have felt the urge to prove their physical capacity in a larger extent than the young adults, simply because they were being observed by the experimenters. Testing their maximum step capability at the start of the experiment might also have contributed to this. Hence, genuine field studies in which the adult’s stepping stones configurations are installed in their environments are needed to test whether these designs foster physical activity in their daily life. Ideally, such a study would compare the spontaneous crossing behavior of older adults in different configurations including the ones that they designed themselves (see for a similar approach Sporrel et al., 2017a).

## Conclusion

The present study demonstrated that older adults are not more conservative in designing their own stepping stone configuration than young adults. Both young and older participants created an non-standardized design with varying gap widths that were challenging for them. In addition, the total distance covered by the older adults was not significantly different from the distance that was traveled by the young adults. These seem to be promising outcomes, especially in light of the public health concern to increase physical activity in seniors. However, field tests are needed to establish whether the older adults’ stepping stones designs invite physical activity in their daily environment.

## Data Availability Statement

All datasets generated for this study are included in the article/supplementary material.

## Ethics Statement

The studies involving human participants were reviewed and approved by the local ethical committee of the Department of Human Movement Sciences, UMCG. The study was approved by the Institutional Ethics Committee (ECB/2016.02.07_1). The patients/participants provided their written informed consent to participate in this study.

## Author Contributions

AJ, RW, and SC dedicated this article to the memory of our graduate student AL who passed away during the final stages of completing this manuscript. All authors contributed to the article and approved the submitted version.

## Conflict of Interest

The authors declare that the research was conducted in the absence of any commercial or financial relationships that could be construed as a potential conflict of interest.

## Footnotes

1 As expected, most participants (44 out of 49) judged their maximum stepping distance larger in the descending trial than in the ascending trial [*t*(48) = -10.20, *p* < 0.001, *d* = 1.46]. Also previous studies, investigating older adults’ perception of maximum forward stepping distance, indicate that an ascending condition results in an underestimation (Almeida et al., 2019) and a descending condition results in an overestimation (Kluft et al., 2017). We therefore averaged across both conditions.

2 As mentioned in the Methods we used the estimated maximum stepping distance in calculating the challenge ratios. Calculating the ratio by dividing the maximum created gap width by the actual maximum step distance does not change the findings. The difference in the actual challenge ratio of the maximum created gap width between young (*M* = 0.77, *SD* = 0.15) and older adults (*M* = 0.79, *SD* = 0.22) is also not significant [*t*(47) = −0.37, *p* = 0.71].

## APPENDIX

**TABLE A1:** Characteristics of the stepping stone configurations created by the young and older adults.

	Participant number	Age (years)	Min gap width (cm)	Mean gap width (cm)	Max gap width (cm)	Total number of gaps^a^	Number of different gap widths^b^	Challenge ratio^c^
Young adults	1	19.9	65	75.8	83	5	2	0.98
	2	20.6	93	100.8	113	6	2	1.08
	3	20.6	91	104.9	115	7	3	0.84
	4	20.1	69	77	86	5	2	0.99
	5	21.5	68	85.6	103	5	3	1.08
	6	21.5	115	123.2	129	6	2	0.94
	7	22.5	58	72.8	92	10	3	1.08
	8	28.3	63	71.4	84	5	3	0.73
	9	22.4	49	78.8	105	9	5	0.86
	10	22.5	45	61.2	82	5	4	0.82
	11	23.0	85	109.5	129	10	4	1.17
	12	22.3	95	100	110	5	2	1.00
	13	21.7	42	55.2	68	10	4	0.78
	14	22.9	76	90.1	106	9	3	1.04
	15	20.5	79	86	93	6	2	1.03
	16	21.4	89	101.4	113	7	3	1.23
	17	26.9	45	53	59	5	3	0.55
	18	28.2	95	105.4	122	5	3	0.96
	19	30.4	70	93.7	104	5	2	0.97
	20	24.6	63	80.5	94	5	4	0.99
	21	28.1	68	73.6	81	5	2	0.60
	22	25.4	5	34.4	70	6	4	0.80
	23	20.6	89	101.9	122	6	3	1.36
	24	20.4	92	96.6	103	5	2	0.79
	25	25.9	56	78.6	95	7	5	0.85
Older	26	71.4	43	80.8	111	7	7	1.23
adults	27	64.1	50	62.5	76	6	3	0.99
	28	75.5	51	55.4	62	5	3	0.81
	29	70.8	41	46.2	56	5	3	0.73
	30	74.0	73	86.6	104	10	3	1.22
	31	70.6	47	64.9	99	6	5	1.08
	32	67.7	41	52.7	64	6	3	0.83
	33	65.3	70	81.5	93	5	2	0.89
	34	64.4	54	65.1	79	6	3	0.81
	35	66.8	43	65.6	83	5	4	0.86
	36	63.3	45	61	78	9	3	0.95
	37	71.3	40	53.8	68	5	3	1.05
	38	77.7	35	48	56	7	4	0.70
	39	70.8	57	71	94	8	4	0.99
	40	74.1	53	66.1	80	7	4	0.87
	41	78.1	37	49.4	67	6	3	0.69
	42	73.8	34	39.8	45	5	3	0.87
	43	74.1	48	57.7	72	6	3	0.85
	44	61.7	87	91	94	6	1	0.97
	45	67.7	71	86.2	107	6	4	1.10
	46	67.2	73	81.3	100	6	3	1.00
	47	63.9	78	81.6	87	5	2	0.74
	48	67.4	35	48	65	10	4	0.61
	49	73.4	11	16.3	20	9	4	0.24

*Only the gaps that were crossed by the participant were considered. ^a^The total number of gaps created (and actually crossed) by participants. ^b^After counting the total number of gaps created (and actually crossed), cluster analysis was executed to calculate the different gap widths within these total numbers of gaps. ^c^Computed by dividing the actual maximum gap width created in the configuration through the maximum estimated gap width determined at the start of the study.*
